# Acute corneal melting one week after an uncomplicated cataract surgery in a patient who previously underwent eyelid radiation and with undiagnosed rheumatoid arthritis: a case report

**DOI:** 10.5935/0004-2749.20210025

**Published:** 2025-02-02

**Authors:** Panagiotis Dervenis, Panagiotis VasilakisPhD, Theodora Stathi, Georgios Giannoulakos, Kiriaki Moula, Nikolaos Dervenis, Anna Praidou, Ioannis Rempapis

**Affiliations:** 1 Department of Ophthalmology, General Hospital of Trikala, Greece; 2 Department of Ophthalmology, AHEPA University Hospital, Thessaloniki, Greece; 3 Department of Ophthalmology, Peterborough City Hospital, Peterborough, UK; 4 Department of Ophthalmology, General Hospital of Thessaloniki “G. Gennimatas - O Agios Dimitrios”, Thessaloniki, Greece

**Keywords:** Corneal perforation, Radiation, Rheumatoid arthritis, Cataract extraction, Perfuração da córnea, Radiação, Artrite reumatoide, Extracão de catarata

## Abstract

This is a rare case report of acute, paracentral corneal melting and perforation occurring 1 week after an uneventful cataract surgery, with discussions on possible pathogenetic mechanisms. Relevant literature was also reviewed. Herein, a case of an 86-year-old woman with acute, paracentral, and sterile corneal melting and perforation in her left eye at 1 week after an uncomplicated cataract extraction is described. This occurs at the base of ocular surface disorders due to previous radiation of her lower eyelid and cheeks for the treatment of cancer and previously undiagnosed rheumatoid arthritis. She underwent surgical treatment using Gundersen’s conjunctival flap for the existing perforation due to low visual expectancies and reluctance to undergo corneal keratoplasty due to the risk of corneal graft rejection. The risk of coming across an acute corneal melting after an uncomplicated cataract surgery in the eyes with ocular surface disorders should always be considered.

## Abstract (pt)

É apresentado um caso raro de ceratomalácia paracentral aguda estéril e perfuração da córnea em uma paciente de 86 anos, uma semana após cirurgia para catarata sem intercorrências. Também são discutidos possíveis mecanismos de patogênese e a literatura relevante é revisada. Esses distúrbios da superfície ocular ocorreram devido à irradiação da pálpebra inferior e da bochecha em um tratamento de câncer e a uma artrite reumatoide não diagnosticada anteriormente. A paciente submeteu-se a um tratamento cirúrgico com um *flap* conjuntival de Gundersen sobre a perfuração existente, devido às suas baixas expectativas visuais e à relutância em submeter-se a uma ceratoplastia da córnea, considerando o risco de rejeição do enxerto corneano. Deve-se sempre considerar o risco de ocorrência de ceratomalácia aguda após cirurgias de catarata sem complicações em olhos apresentando distúrbios da superfície ocular.

## INTRODUCTION

Cataract is a major health problem in people aged >50 worldwide, and thus, cataract surgery is the most frequent surgical procedure performed nowadays. Advances in surgical techniques and instrumentation have greatly limited the occurrence of postoperative complications^(1)^.

Corneal melting is an unusual complication of phacoemulsification. Several predisposing risk factors have been associated with this complication, such as dry eye disease, rheumatoid arthritis, topical nonsteroidal anti-inflammatory drugs (NSAIDs), and corneal infections^(2-5)^.

Orbital and periocular radiation has been reported to have a major effect on tear film stability and ocular surface, inducing decreased corneal sensitivity, dry eye problems, and neurotrophic keratopathy^(6,7)^.

Rheumatoid arthritis and other collagen vascular diseases have been known to affect the cornea. Although peripheral corneal ulceration is the most common corneal manifestation of rheumatoid arthritis, central and paracentral corneal ulceration and perforation may also occur^(8)^. These ulcers often appear with quiescent systemic arthritis^(9)^.

Lastly, corneal melting is the most serious side effect of topical NSAIDs. Although several controversial studies have been reported through the years, NSAIDs-induced corneal melting has been reported by several researchers^(4)^.

To our best knowledge, this is the first case report describing an acute corneal melting after phacoemulsification as a first symptom of an otherwise quiescent, undiagnosed rheumatoid arthritis in a patient with eyelid radiation history.

## CASE REPORT

An 86-year-old woman was referred to our clinic due to a progressively blurring vision in her left eye. She had a history of two courses of External Beam Radiotherapy (EBRT) in her left eyelid and cheek for the treatment of basal cell carcinoma. The first treatment was performed 13 years ago and the second one was performed 3 years before the cataract surgery. Besides that, her past medical history was unremarkable.

The anterior segment examination revealed lid margin irregularity, vascular engorgement, few plugged meibomian gland orifices, and mucotaneous junction displacement in her left lower eyelid. Otherwise, it was unremarkable, showing the presence of moderate nuclear sclerosis in her left eye and no signs of dry eyes. Her best-corrected visual acuity (BCVA) was 6/15. The remaining clinical examination, including dilated fundoscopy and IOP (Intraocular Pressure) measuring, revealed no other pathologies in her both eyes.

After obtaining an informed consent, she underwent uncomplicated cataract extraction with posterior chamber IOL (Intraocular Lens) implantation in her left eye. Postoperatively, the treatment regimen included administration of 0.5% chloramphenicol/0.1% dexamethasone eye drops four times daily and 0.9 mg/ml Bromfenac twice daily. The postoperative use of NSAIDs is a common clinical practice in our clinic due to its confirmed ability to reduce the risk of Irvine-Gass syndrome^(10)^.

**Figure 1:**
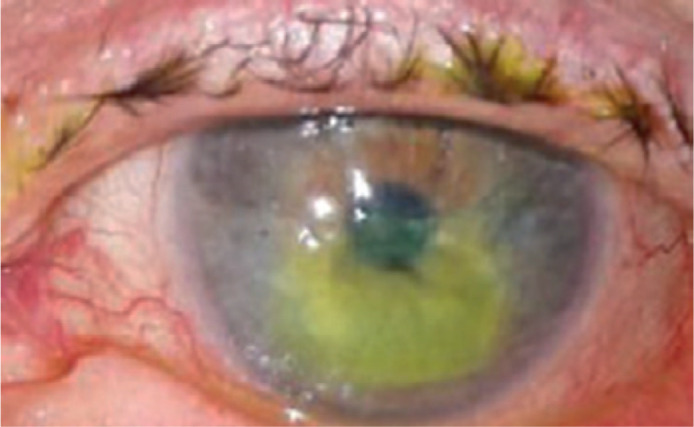
Acute corneal melting.

One week later, she presented with a painless, paracentral area of sterile corneal melting of 4 mm in diameter and a central perforation area of 3 mm in diameter. (Figure 1) The melting was non-infiltrated and far from the incision site. She had a flat anterior chamber with a positive Seidel test, and her BCVA was hand movement.

She was immediately admitted and treated with levofloxacin 0.5% eye drops (q1h) and intravenous cefuroxime. Intravenous was preferred over intravitreal administration because there was no sign of acute endophthalmitis and it was crucial to prevent inoculation of microorganisms occurring from contiguous ocular structures inside the eye. In this way, superimposed bacterial infection and possible underlying systemic infection were also excluded. Microbiological examinations were not performed because signs of infection were not observed. Blood tests were requested, including erythrocyte sedimentation rate, complete blood count with differential, rheumatoid factor, antinuclear antibody, antineutrophilic cytoplasmic antibody levels, angiotensin-converting enzyme, and chest x-ray. Based on these test results, rheumatoid factor and antinuclear antibodies have been identified, and mild increase in ESR (Erythrocyte Sedimentation Rate) and mild thrombocytosis were observed. Afterward, the patient was diagnosed with rheumatoid arthritis by our rheumatologists in accordance with the 2010 American College of Rheumatology (ACR)/European League Against Rheumatism classification criteria, which she controlled with 2.5 mg of methotrexate (three tabs two times per week) and 5 mg of prednisolone (two tabs per day). They opted not to use intravenous steroids because of her quiescent arthritis.

**Figure 2:**
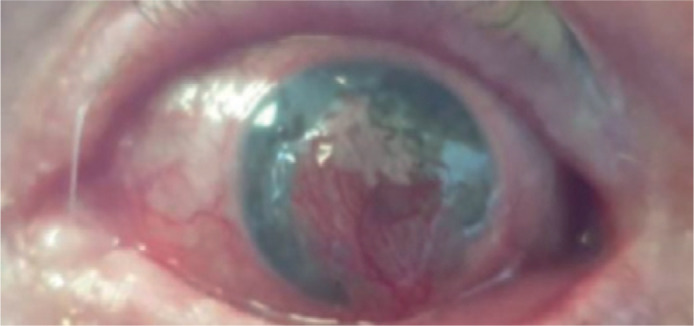
Six months postoperatively.

Due to the urgency of the incidence, we decided to cover the perforation area with Gundersen’s conjunctival flap due to the absence of limbal vasculitis, low visual expectancies, and her unwillingness to undergo keratoplasty. One day postoperatively, the anterior chamber was formed, Seidel test was negative, and no signs of infection or corneal melting were observed. Six months later, the integrity of her anterior chamber has been achieved and no recurrences occurred, whereas her BCVA was 6/60 (Figure 2).

## DISCUSSION

In this report, we describe the case of a patient presenting with an acute corneal melting and perforation, one week after an uneventful phacoemulsification. In our patient, we believe that the urgent progress from corneal melting to perforation was multifactorial: exacerbation of an undiagnosed post-radiation tear film dysfunction, undiagnosed rheumatoid arthritis, and NSAID treatment.

Periocular and orbital radiations have been associated with tear film instability. Radiation greatly affects the meibomian gland functionality^(7)^ and induces morphological and functional loss of lacrimal glands^(6)^. Moreover, experimental studies have introduced the term radiation keratopathy as a result of corneal nerve loss and increased influx of immune cells (CD45+) in the cornea,^(11)^ engendering tear film dysfunction due to reduced reflex tearing and corneal sensitivity. This disruption of the sensory pathway can also induce neurotrophic keratitis^(12)^. Lastly, studies have shown that a preexisting mild tear film instability can exacerbate postoperatively^(13)^ and induce corneal melting^(5)^. In addition, studies have shown that approximately 50% of patients with tear film dysfunction are asymptomatic during preoperative clinical examination^(14)^.

Rheumatoid arthritis can dramatically affect a human’s cornea in two different ways: peripheral ulcerative keratitis (PUK) and central/paracentral keratolysis. PUK occurs due to local imbalance between the collagenase MMP-1 concentration and its inhibitor, TIMP-1^(15)^, as a result of an immune microangiopathy and inflammatory mediator leakage that is present in the limbus. An aberrant cell-mediated response to epithelial damage, secondary to an irregular expression of HLA-II antigens in the corneal epithelium, has also been proposed^(16)^. The absence of limbal vasculitis distinguishes paracentral keratolysis from PUK. Apart from the irregular HLA-II antigen expression, IgG and IgM accumulation occurred in the corneal epithelium and T-cell infiltrating the stroma. The main associated molecules are CD-11c and CD3. Moreover, an antibody has been found to react against myeloperoxidase of polymorphonuclear white blood cells. The mechanism of inflammation is an epithelial barrier dysfunction that allows immune complexes entering the stroma and provoking keratolysis^(8,9,16)^.

Finally, recent studies have shown a correlation between NSAIDs and corneal ulceration^(4)^. Different mechanisms have also been proposed, including metalloproteinase activation, impaired wound healing, and altered neurotrophic effect due to analgesia^(17)^. Although nepafenac and ketorolac have been primarily associated with sterile ulceration, other reports also demonstrated Bromfenac having the same effect^(18)^.

Concerning the treatment of our patient, Gundersen’s conjunctival flap was the golden section of ensuring globe’s integrity and reluctance to undergo keratoplasty with guarded prognosis^(16)^. The absence of limbal and conjunctival vasculitis excluded conjunctival resection from our options, because it has been confirmed to have no therapeutic effect^(8)^. Finally, the perforation size made the use of cyanoacrylate glue impossible.

To our best knowledge, no other cases have been reported on sterile corneal perforation after a cataract surgery in a periocularly irradiated patient as the first symptom of a previously undiagnosed rheumatoid arthritis. Therefore, we believe that ocular surface disor ders caused by a previous radiation, undiagnosed rheumatoid arthritis, and use of NSAIDs were predisposing factors associated with this complication. This case report increases the awareness on this sight-threatening complication following a cataract surgery. Thorough clinical examination and systemic investigation should be performed in patients who are highly clinically suspected.
